# Association of *LRP1B* Mutation With Tumor Mutation Burden and Outcomes in Melanoma and Non-small Cell Lung Cancer Patients Treated With Immune Check-Point Blockades

**DOI:** 10.3389/fimmu.2019.01113

**Published:** 2019-05-21

**Authors:** Hao Chen, Wei Chong, Qian Wu, Yueliang Yao, Min Mao, Xin Wang

**Affiliations:** ^1^Clinical Epidemiology Unit, Qilu Hospital of Shandong University, Jinan, China; ^2^Key Laboratory of Cancer Prevention and Therapy of Tianjin, Department of Epidemiology and Biostatistics, National Clinical Research Center for Cancer, Tianjin Medical University Cancer Institute and Hospital, Tianjin, China; ^3^Key Laboratory of Cancer Prevention and Therapy, Department of Breast Cancer Pathology and Research Laboratory, National Clinical Research Center for Cancer, Tianjin Medical University Cancer Institute and Hospital, Tianjin, China; ^4^Department of Respiratory Medicine, Central Hospital of Zibo, Zibo, China; ^5^Institute of Pathology and Southwest Cancer Center, Southwest Hospital, Army Medical University (Third Military Medical University), Chongqing, China; ^6^Key Laboratory of Tumor Immunopathology, Ministry of Education of China, Institute of Pathology and Southwest Cancer Center, Southwest Hospital, Third Military Medical University (Army Medical University), Chongqing, China; ^7^Department of Epidemiology and Biostatistics, First Affiliated Hospital, Army Medical University, Chongqing, China

**Keywords:** *LRP1B*, melanoma, NSCLC, tumor mutation burden, immunotherapy, mutation signatures

## Abstract

**Background:** Tumor mutation burden (TMB) have been served as the most prevalent biomarkers to predict immunotherapy response. *LRP1B* (low-density lipoprotein receptor-related protein 1B) is frequently mutated in melanoma, non-small cell lung cancer (NSCLC) and other tumors; however, its association with TMB and survival in patients with immunotherapy remains unknown.

**Methods:** We curated somatic mutation data and clinicopathologic information from 332 melanoma immunotherapy samples for discovery and 113 NSCLC samples for further corroboration. Bayesian variants non-negative matrix factorization was used to extract tumor mutational signatures. Multivariate Cox and logistic regression models were applied to adjust confounding factors. The CIBERSORT and GSEA algorithm were separately used to infer leukocyte relative abundance and significantly enriched pathways.

**Results:** Patients with *LRP1B* mutation were identified to be associated with prolonged survival in both immunotherapy cohort. Higher tumor mutation burden was found in *LRP1B* mutated patients, and the association remained significant after controlling for age, gender, stage, mutations in *TP53* and *ATR*, and mutational signatures. Immune response and cell cycle regulation circuits were among the top enriched pathways in samples with *LRP1B* mutations.

**Conclusion:** Our studies suggested sequencing even a single, frequently mutated gene may provide insight into genome-wide mutational burden, and may serve as a biomarker to predict immune response.

## Introduction

Immune checkpoint blockades (ICB) therapy such as anti-CTLA-4 and/or anti-PD-1 have demonstrated durable antitumor effects in treatment of multiple cancers (1–4). Tumor mutation burden was broadly considered as biomarkers associated with clinical response to ICB treatment in melanoma (5–7), non-small cell lung cancer (NSCLC) (8, 9), colorectal and gastric cancers (10). Mutations in genomic integrity associated genes, such as *TP53* (11) and *ATR* (12), could cause genomic instability, replication stress and resulting a higher mutation rate in tumor genomes. Somatic mutation caused tumor-specific neoantigen (neopeptide fragments) that could serve as markers to identify the responders to ICB treatment (13, 14). It is also reported that neoantigen burden have a correlation with tumor mutation burden (5, 8).

The low-density lipoprotein receptor-related protein 1B (*LRP1B*), which encoding endocytic LDL-family receptor, is among the top 10 significantly mutated genes in human cancer (15). It has been demonstrated that *LRP1B* could bind to multiple extracellular ligands, including fibrinogen and apoE-carrying lipoproteins. Frequently inactivation mutation of *LRP1B* was observed in melanoma (16), lung cancer (17), esophagus squamous-cell carcinoma (18), head and neck squamous cancer (19, 20), gastric cacner (21), and so on. Owing to its large size (coding sequence, 16 kbp), *LRP1B* is often missed as a significantly mutated gene analysis, whereas its mutation still could have a functional consequence in tumorigenesis and heterogeneity.

The characteristic mutational signatures are the fingerprints of endogenous and exogenous factors that have acted over the course of tumor development and progression. Exogenous mutational signatures, such as ultraviolet radiation exposure (22, 23), tobacco smoking (9) and endogenous mutational signatures, such as *APOBEC* family of cytidine deaminases (24) mismatch repair defiency (10) were all contributed to higher tumor mutation burden and immune responding.

Tumor microenvironment (TME) also associate with response to ICB therapy. Baseline levels of tumor-infiltrating CD8^+^ T cells, CD4 T^+^ cells and NK cells were shown to be correlated with the likelihood of immune response (25–27). Mutations in genes involved in antigen presentation and interferon-related circuits were reported with immune (28, 29). Recently, a T cell-inflamed gene expression profile (GEP) was shown to predict response to immunotherapy (30, 31).

*LRP1B* is one of the most frequently mutated genes in tumor samples; however, its associations with TMB and prognosis remain unclear. In this study, we investigated whether *LRP1B* mutations are associated with TMB and survival prognosis in patients treated by immune check-point blockades. Findings emerged from this study may be useful for guiding immunotherapy treatment for cancer patients.

## Methods

### Genomic Data and Clinical Information of Melanoma and NSCLC

Somatic mutations were acquired from previous WES studies totaling 332 melanoma cases (5–7, 23, 34) and 113 NSCLC cases (8, 9, 32). Whole exome capture libraries were constructed using the Agilent SureSelect All Exon V2/V4 or 50 Mb kit. Enriched exome libraries were sequenced on a HiSeq 2000, 2500, or 4000 platform (Illumina) to generate paired-end reads (2 × 76 bp/100 bp) to a goal of 178X mean target coverage (range 32–380). The detailed sequencing information of each cohort was collected and illustrated in Supplementary Table S1. Mutations were re-annotated by oncotator against hg19 reference genome (33). Clinicopathological information including age, gender, stage, PD-L1 expression, smoke, ICB types, immune response status and survival were curated from supplemental materials of these studies (Supplementary Table S2). The predicted MHC binding affinity scores, HLA types, and clinical features were also collected from the previous studies (5, 7–9, 32, 34) (melanoma, *n* = 224; NSCLC, *n* = 113). Patients with complete or partial responses were considered to be efficacious to ICB treatment and the rest were regarded as the non-responding. Gene expression data in melanoma were available in Riaz.N and Hugo.W cohorts (6, 23). Gene expression data for NSCLC cohort were not available. Somatic mutations for samples in the TCGA datasets of SKCM (*n* = 467), NSLC (*n* = 998) were downloaded from Genome Data Commons (https://portal.gdc.cancer.gov).

### Deciphering Mutational Signature Operative in the Genome

We used SignatureAnalyzer (https://software.broadinstitute.org/cancer/cga/Home) proposed by Kim et al. (35) to extract mutational signatures from the aggregated somatic mutation data in melanoma and NSCLC cohorts. This framework is based on Bayesian variant non-negative matrix factorization and it can automatically determine the optimal number of extracted mutational signatures. The SignatureAnalyzer factorized the mutation portrait matrix into two non-negative matrices ***W*** and ***H***, where ***W*** representing mutational processes and ***H*** representing the corresponding mutational activities. The number of mutational signatures is the number of columns of matrix W. The rows of matrix A are the 96 mutational contexts (i.e., C > A, C > G, C > T, T > A, T > C, T > G, and their 5′and 3′ adjacent bases), and its columns are the samples of each cohorts (melanoma, *n* = 332; NSCLC, *n* = 113). Mutational signatures were annotated by calculating cosine similarity against 30 validated mutational signatures in the Catalog of Somatic Mutations in Cancer (COSMIC, v85) (36).

### *LRP1B* Mutation With Tumor Mutation Burden

The extracted mutational signatures were stratified as binary variables (i.e., 0 and 1) in the multivariate model. The classified method is according to the previous study, which a signature was considered significant if it contributed to more than 100 substitutions or more than 25% of total mutation activities (37). As mutations in *TP53* and *ATR* and some mutational signatures increase mutation rates in the cancer genome, we used Generalized Linear Models and Fit Proportional Hazards Regression Model to analyze associations between *LRP1B* mutation and TMB by including them as confounding factors. TMB was defined the number of non-synonymous alterations (SNVs or indels) using whole exome sequencing for each patient, and the median served as the cutoff value of high vs. low.

### Gene set Enrichment Analysis

We partitioned 72 cases whose gene expression profiles were available into two groups according to mutation status of *LRP1B*. Specifically, read counts of gene expression data were normalized by calcNormFactors in R package edgeR and remove batch effect by removeBatchEffect functions in package limma. We then fed them into lmFit and eBayes functions in the R limma package, and used these statistics as input to R-function in ClusterProfile package to do gene set enrichment analysis (GSEA). The gene sets examined in GSEA of REACTOME pathways were obtained from MSigDB database (v6.2) (38).

### Tumor Infiltrating Lymphocyte Cell Analysis

We curated gene expression profile of 72 pre-treatment melanoma samples from two studies of Riaz and Hugo et al. (6, 23). The relative abundance of 22 tumor infiltrating lymphocyte cells (TILs) in different *LRP1B* mutation status were estimated by the CIBERSORT algorithm (39), a computational approach for inferring leukocyte representation in bulk tumor transcriptomes.

### T Cell-Inflamed Gene Expression (RNA) Profiling (GEP) Scores

We applied and followed the T cell-inflamed Gene expression (RNA) profiling (GEP) proposed by Ayers et al. (31) to quantify the GEP scores. The GEP was composed of 18 inflammatory genes associated with chemokine expression, cytolytic activity, antigen presentation, and adaptive immune resistance, including *CD274 (PD-L1), CD276 (B7-H3), CCL5, CD27, CD8A, CMKLR1, CXCL9, CXCR6, IDO1, LAG3, NKG7, PDCD1LG2 (PDL2), PSMB10, HLA-DQA1, HLA-DRB1, HLA-E, STAT1*, and *TIGIT*. GEP scores were calculated as a weighted mean of normalized expression values for the 18 genes.

### Statistical Analyses

The statistical analyses in this study was performed with R software (version 3.2.3). The continuous variables between groups were compared by the Wilcoxon rank sum test. The association between proportion of mutated genes and immune therapy response was evaluated by Fisher's exact test. Kaplan-Meier survival analysis and Cox proportional hazards model were used to analyze the association between mutated genes and prognosis with the R package *survminer* and *forestmodel*. Association of *LRP1B* mutation with TMB was examined by the logistic regression by including confounding factors such age, gender, stage, *PD-L1, TP53, ATR* mutation and extracted mutational signatures. All comparisons were two-sided with a significance level of 0.05, and the Benjamini-Hochberg method was applied to control false discovery rate (FDR) for multiple hypothesis testing (40).

## Results

### Tumor Genomic Alterations in Melanoma Cohort

**Figure 1:**
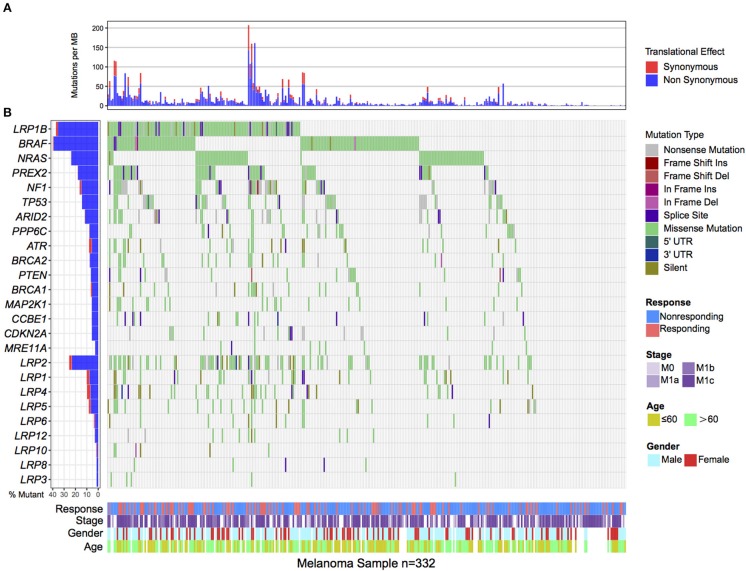
Mutational patterns of recurrently mutated melanoma genes, Genomic Instability associated genes and LRPs gene family in relation to *LRP1B* mutation in the pooled melanoma patients. **(A)**. Mutation rates per megabase stratified by synonymous and non-synonymous mutations. **(B)**. The left panel is mutation frequency and the middle panel depicts genes mutation patterns across each cases with different mutation types color coded differently. Clinical features of immune response status, age, gender and stage displays in bottom. Immune response related genes were highlighted in bold.

Of the 332 melanoma patients from previous genomic immune studies, 98 (29.5%) were recognized as immune responders. *LRP1B* was one of the most frequently mutated genes in the aggregated melanoma cohort, accounting for 122 of 332 patients (36.7%). The mutation plots of *LRP1B* in different immune response status are shown in Supplementary Figure 1. Melanoma samples with *LRP1B* mutations had higher mutation rate than samples without *LRP1B* mutation (Figure 1A). The well-known melanoma driver oncogenes (e.g., *BRAF, NRAS, NF1, PREX2, ARID2, PPP6C, CCBE1, PTEN*), genomic integrity maintenance and DNA replication proofreading associated genes (e.g., *TP53, ATR, BRCA1/2, MRE11A*) and common LRPs (i.e., *LRP1, LRP2, LRP3, LRP4, LRP5, LRP6, LRP8, LRP10*, and *LRP12)* in relation to *LRP1B* mutation were illustrated in waterfall plot (Figure 1B). Mutations in *LRP1B, MRE11A* were correlated with improved immune response (Fisher's exact test, *P* < 0.05; Figure 1B).

### *LRP1B* Mutation Predictive of Immunotherapy Survival in Melanoma

**Figure 2:**
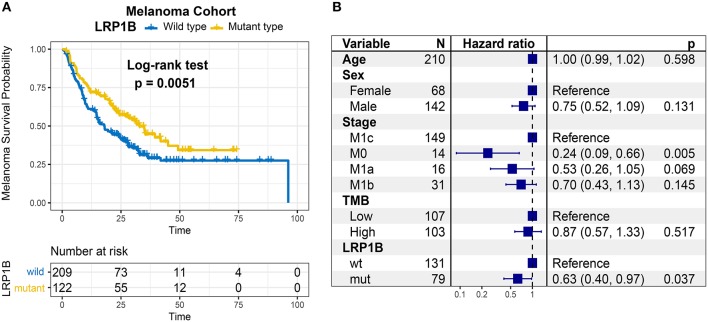
Association of *LRP1B* Mutation With Prognosis in melanoma immunotherapy Cohort. **(A)** Kaplan-Meier survival analysis classified by *LRP1B* mutation status. **(B)** Multivariate Cox regression analysis of *LRP1B* mutations with age, gender, TMB status, TNM stage were taken into account.

In Kaplan-Meier survival analysis, the *LRP1B* mutation was significantly associated with a better immunotherapy survival outcome in the pooled melanoma cohort (log-rank test, *P* = 0.005; Figure 2A). The association between *LRP1B* mutation with survival remained statistically significant after taking into account age, gender, TMB status and stage (Cox proportional hazards model, HR, 0.63 [95%CI, 0.40–0.97], *P* = 0.037; Figure 2B). We also noticed a preferable immune response status in *LRP1B* mutant samples (Fisher's exact test, *P* = 0.008).

### *LRP1B* Mutation Associated With TMB

**Figure 3:**
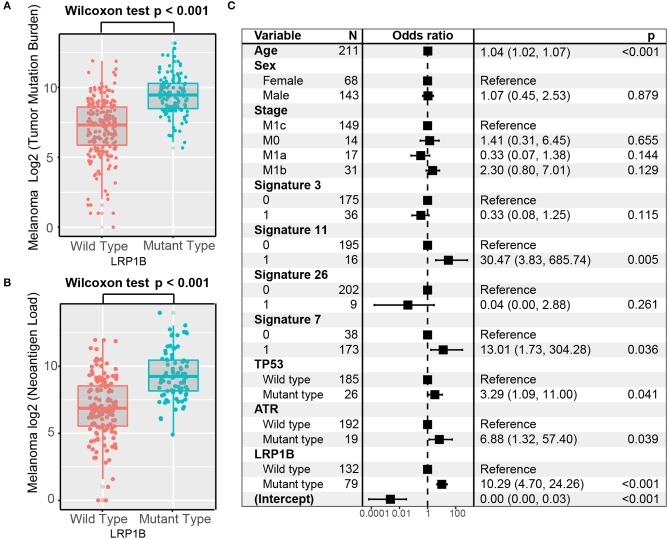
Association of *LRP1B* Mutation With Higher Tumor Mutation Burden in pooled melanoma cohort. Tumor mutation burden **(A)** and neoantigen burden **(B)** of melanoma stratified by *LRP1B* mutation status. **(C)** Multivariate Logistic regression analysis of *LRP1B* mutation with TMB after adjusted for age, sex, TNM stage, mutational signatures, and mutations in TP53 and ATR. Square data markers indicate estimated hazard ratios. Error bars represent 95% CIs.

Melanoma samples with *LRP1B* mutations had a significantly higher tumor mutation burden and neoantigen burden by Wilcoxon rank sum test (log_2_ TMB, 9.5 vs. 7.3, *P* < 0.001; log_2_ NB, 9.2 vs. 6.9, *P* < 0.001; Figures 3A,B). We also observed the parallel result in TCGA melanoma cohort (log_2_ TMB, 9.2 vs. 7.7; Wilcoxon rank sum test, *P* < 0.001; Supplementary Figure 2). Tumor mutation burden is largely attributed to genomic instability, which is prevalent in melanoma. In these samples, we extracted 6 mutational signatures (Supplementary Figures 3A,B), including signatures related to genomic instability. The numbers of somatic mutations attributed to each mutational signature varied considerably in each sample. Underlying associations with these mutational signatures included failure of DNA double-strand break-repair by homologous recombination (signature 3, 8,745 of 207,310, 4.2%), defective DNA mismatch repair (signature 26, 3,050 of 207,310, 1.5%), alkylating agent treatment (signature 11, 30,835 of 207,310, 14.9%) and ultraviolet light exposure (signature 7, 157,250 of 207,310, 75.9%). Samples with signature 7 and signature 11 had greater TMB compared with samples without these features (Supplementary Figure 3C). The mutational activity attributable to each mutational signature in each melanoma sample and variation were shown in Supplementary Table S3.

To rule out the possibility that associations between *LRP1B* mutations and TMB were affected by these confounding factors, we included mutation associated signatures (3, 7, 11, 26) and mutations in *TP53* and *ATR* in the multivariate model. Associations between *LRP1B* mutations and TMB remained statistically significant after adjustment (logistic regression model, OR, 10.29; 95% CI, 4.70–24.26, *P* < 0.001; Figure 3C).

### Further Corroboration of *LRP1B* Mutations in the NSCLC Cohort

**Figure 4:**
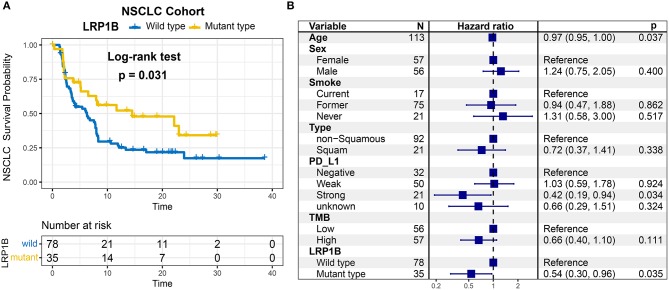
*LRP1B* mutation is associated with immunotherapy survival outcome of NSCLC patients. **(A)** Kaplan-Meier survival analysis stratified by *LRP1B* mutation. **(B)** Multivariate Cox regression analysis of *LRP1B* mutation by taking into account confounding factors, such as age, gender, PD-L1 expression, TMB, smoke status, histology type.

As *LRP1B* mutation was common in lung cancer, we aggregated whole exome sequencing data from three studies of NSCLC immunotherapy to corroborate the results. *LRP1B* was also frequently mutated (35 of 113 patients [31.0%]) in the pooled NSCLC cohort. The mutation plots of *LRP1B* in NSCLC of different immune response status are shown in Supplementary Figure 4. Mutation in *LRP1B* was significantly associated with a better survival prognostic (log-rank test, *P* = 0.031; Figure 4A) even after taking into account age, gender, smoke, TMB and PD-L1 expression status (Cox proportional hazards model, HR, 0.54 [95%CI, 0.30–0.96], *P* = 0.035; Figure 4B).

**Figure 5:**
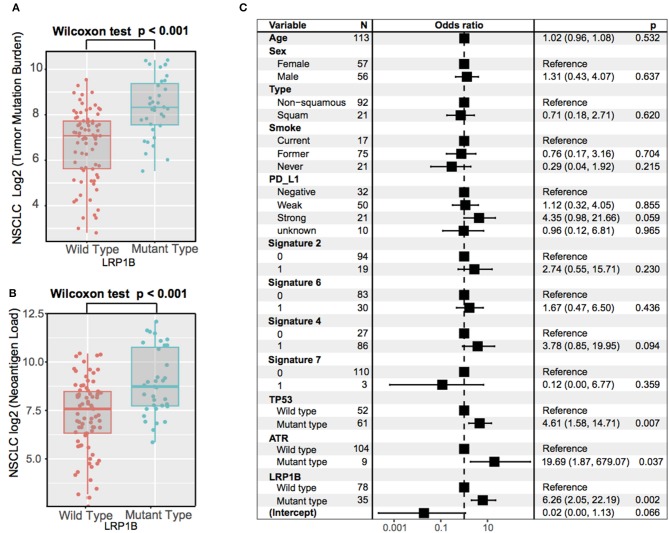
Association of *LRP1B* Mutation With Higher Tumor Mutation Burden in aggregated NSCLC cohort. Tumor mutation burden **(A)** and neoantigen burden **(B)** of NSCLC stratified by *LRP1B* mutation status. **(C)** Multivariate Logistic regression analysis of *LRP1B* mutation with TMB after adjusted for age, sex, PD-L1, smoke status, mutational signatures, and mutations in *TP53* and *ATR*. Square data markers indicate estimated hazard ratios. Error bars represent 95% CIs.

The significantly higher tumor mutation burden and neoantigen burden were observed in aggregated NSCLC samples with *LRP1B* mutations by Wilcoxon rank sum test (log_2_ TMB, 8.3 vs. 7.1, *P* < 0.001; log_2_ NB, 8.7 vs. 7.4, *P* < 0.001) (Figures 5A,B). The similar results was also observed in TCGA NSCLC cohort (log_2_ TMB, 8.3 vs. 7.4; Wilcoxon rank sum test, *P* < 0.001, Supplementary Figure 5). The extracted mutational signatures in NSCLC samples included signature 4 (tobacco smoking), signature 2 (*APOBEC* family of cytidine deaminases), signature 6 (DNA mismatch repair), signature 7 (UV exposure) and signature 16 (etiology unknown) (Supplementary Figures 6A,B). The mutational activity attributable to each mutational signature in NSCLC sample were shown in Supplementary Table S4. NSCLC samples with signature 2 and signature 4 had greater TMB compared with samples without these features (Supplementary Figure 6C). Associations of *LRP1B* mutations with the lung cancer driver oncogenes (*KRAS, STK11, EGFR, ROS1, ALK, PTEN, CCBE1*), maintaining genomic integrity genes (*TP53, ATR, POLE*), and LRPs gene family are shown in the middle panel of Supplementary Figure 7. The association of *LRP1B* mutations with higher TMB remained statistically significant after controlling for age, gender, smoke, PD-L1 expression, mutational signatures (etiology known), and mutations in *TP53* and *ATR* in the multivariate model (Logistic regression model, OR, 6.26 [95%CI, 2.05–22.19], *P* < 0.001; Figure 5C).

We also analysis the association between binding affinity (IC50) of candidate neoantigen and clinical benefit, and observed a significantly lower value in immune responder group (median binding affinity, melanoma: 149 vs. 174, *p* < 0.001; NSCLC: 163 vs. 169, *p* = 0.006). Besides, a decreased predicated binding affinity (IC50) were also detected in *LRP1B* mutant samples, although these difference was not statistically significant (median binding affinity, melanoma: 166 vs. 170, *p* = 0.168; NSCLC: 164 vs. 167, *p* = 0.392). This is also the case in predicated neoantigen peptides which caused by LRP1B mutation (median, melanoma: 181 vs. 199, *p* = 0.293; NSCLC: 124 vs. 167, *p* = 0.079).

### Significantly Enriched Pathways and Immunocytes Associated With *LRP1B* Mutations

**Figure 6:**
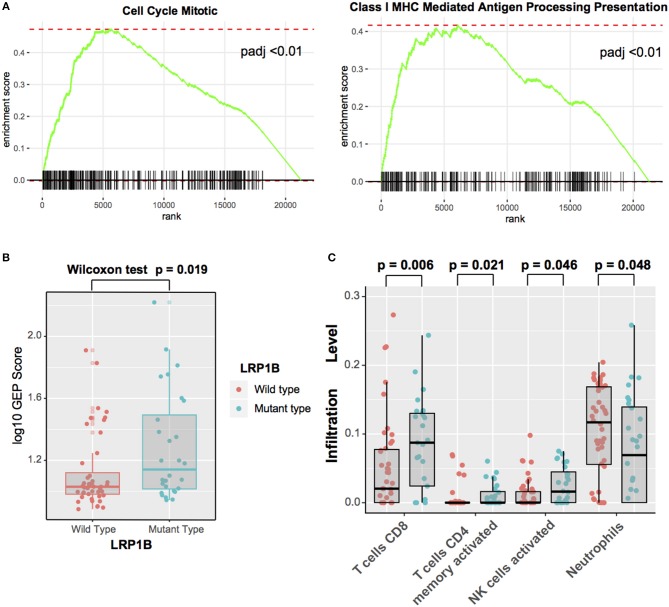
Significantly Enriched Pathways and Immunocytes Associated With *LRP1B* Mutations **(A)** Top enriched Reactome pathways in *LRP1B* mutant group vs. *LRP1B* wild-type group. **(B)** T cell-inflamed GEP with *LRP1B* mutation was assessed in melanoma. **(C)** Relative abundance of tumor infiltrating leukocytes in *LRP1B* mutant vs. *LRP1B* wild-type samples.

As *LRP1B* play an important role in immunotherapy outcomes, we investigate the potential mechanism behind *LRP1B* mutation and immune response. Gene set enrichment analysis (GSEA) on Reactome gene sets revealed enrichment of genes involved in Cell Cycle Mitotic and Antigen Processing and Presentation pathways were significantly altered in samples with *LRP1B* mutations (*p.adj* < 0.001, Figure 6A, Supplementary Figure 6B). Nevertheless, Complement Cascade, Formation of Tubulin Folding were enriched in wild-type group (*p.adj* < 0.001, Supplementary Figure 8). Besides, T cell-inflamed gene expression profile (GEP) were also analyzed and found a higher scores in *LRP1B* mutational groups (GEP scores, 1.14 vs. 1.03, Wilcoxon rank sum test, *P* = 0.019; Figure 6B).

Moreover, we evaluated (with use of CIBER algorithm) the abundance of tumor-infiltrating lymphocyte cells in melanoma microenvironment using the gene expression data. We found that CD8^+^ T cells, activated CD4 memory T cells, activated NK cells, were more enriched in *LRP1B* mutant type group, nevertheless, Neurtrophils was enriched in wild-type group (Figure 6C).

## Discussion

We analyzed *LRP1B* mutation with immune response and outcome in 332 samples from the melanoma immunotherapy cohort and 113 samples from the NSCLC immunotherapy cohort for further corroboration. *LRP1B* was frequently mutated in melanoma and NSCLC, and its mutation was associated with higher TMB and better survival outcome. The association of *LRP1B* mutation with TMB was independent of a significant presence of mutational signatures and of mutations in *TP53* and *ATR*. Samples with *LRP1B* mutations were characterized by upregulation of signaling pathways involved in immune system, antigen processing and presentation and cell cycle check-points. Tumor-infiltrating immune cells results manifested *LRP1B* mutated samples were infiltrated in CD8^+^ T cells, NK cells and activated CD4 memory T cells, which supported the previous observations that such leucocytes and pathways present predominately in the tumor microenvironment of immune responder and promote the immune response (27, 41).

Besides melanoma and non-small cell lung cancer, *LRP1B* was also frequently mutated in multiple types of human cancer. Several observations favor *LRP1B* being a *bona fide* driver mutation in many tumors, including a very high frequency of homozygous deletions (42), and frequent point mutation in esophagus (18), oral (20), liver (43), colon (44), gastric (21), breast (45), thyroid (46), and pancreatic cancer (47). Recently, *LRP1B* has been implicated in antigen presentation and as a regulator of inflammation and progression in cancer (47, 48). Owing to its large size, *LRP1B* was often excluded from lists of significantly mutated genes. Another common ignored gene was *MUC16*, but Li et al. found *MUC16* mutation was strongly associated with tumor mutation load and prognostic outcome in gastric cancer patients, and may benefits for patients with immunotherapy (49). The mechanisms underlying the association between TMB and *LRP1B* is not entirely clear. A leading hypothesis suggests that the large size (coding sequence,16 kbp) and gene location near the FRA2F fragile site (50) may contribute to a higher tumor mutation rate.

The classical role of LRPs is to modulate the clearance of a numerous of extracellular ligands from the pericellular microenvironment (51, 52). In recent years, LRPs emerged as an important regulator of the inflammatory response. *LRP1* was supposed to modulate the microglial immune response via regulation of c-Jun N-terminal kinase (JNK) and NF-κB signaling pathways (53). Overexpression of *LRP1* is associated with worsened prognosis and suppressive tumor immunity in renal clear-cell carcinoma (54). Deletion of *LRP5* and *LRP6* in dendritic cells markedly delayed tumor growth and enhanced host antitumor immunity by blocking the Wnt pathway (55). Two SNPs in atopy-related immunologic candidate genes *LRP1B* were associated with pancreatic cancer risk, even after adjustment for multiple comparisons (47).

Recent studies also revealed *LRP1B* may as a tumor suppressor in common chemotherpay with deletion mutation of *LRP1B* causing a chemoresistance and poor outcome (56, 57). In TCGA database, patients with *LRP1B* mutation have no benefits on conventional chemotherapy in melanoma and NSCLC (Supplementary Figure 9), but displayed preferable clinical outcome in immune check-point blockades therapy, suggested the molecular marker prediction of selectivity and specificity in drug treatment.

The main limitation is using the public dataset from different cohort which are somewhat heterogeneous in data processing and patient population. Although we utilized multiple datasets in different tumor type for analysis, but lack of the independent dataset for validation in the same tumor types. The analyzing tools used in sequencing data may have been different between these studies and may introduce bias in the final mutation lists. Besides, the number of samples with expression data in the cohort was limited, which limits the ability to adjust statistical power. As a result, association data between mutational patterns and gene expression, including analysis in immune cell infiltration and oncogenic pathways need further validation and studies.

Our studies suggested that sequencing even a single, frequently mutated gene may provide insight into genome-wide tumor mutational burden and could be served as a biomarker to predict immune response. The mechanisms through which *LRP1B* induce high mutations rate in tumors are still unknown and require future investigation. An investigation of a possible role of *LRP1B* in response to immune check-point blockade treatment in other cancer types (HNSC, ESCC, STAD, etc.) is warranted to study. The full implication of *LRP1B* in immunotherapy prognostic and monitoring remains elusive and requires in-depth studies.

## Data Availability

All relevant data and materials within this work are made available in this manuscript and TCGA databases.

## Ethics Statement

All studies have been approved by the Institutional Research Board.

## Author Contributions

All authors reviewed the manuscript and agreed to submission. HC, QW, and XW designed the project. HC, QW, MM, WC, and YY performed administrative, technical, or material support. HC and XW performed statistical analysis. HC and WC wrote the manuscript. XW and MM revised the paper.

### Conflict of Interest Statement

The authors declare that the research was conducted in the absence of any commercial or financial relationships that could be construed as a potential conflict of interest.

## Supplementary Material

Supplementary Figure 1 Lollipop plot shows the distribution of *LRP1B* mutations in melanoma responder cohort and non-responder cohort.

Supplementary Figure 2 Tumor mutation burden(TMB) associated with *LRP1B* mutation status in TCGA skin melanoma database.

Supplementary Figure 3 Mutational signatures extracted from the aggregated melanoma dataset. **(A)** The mutational activities of corresponding extracted mutational signatures (Signature 1, 3, 7, 11, 18, 26, named as COSMIC signature). **(B)** The mutational activities of corresponding mutational signatures showed in pie chart. **(C)** The mutational signature 7 and 11 in melanoma cohort was associated with higher tumor mutation burden.

Supplementary Figure 4 Lollipop plot shows the distribution of *LRP1B* mutations in non-small cell lung cancer responder and non-responder cohort.

Supplementary Figure 5 Tumor mutation burden(TMB) associated with *LRP1B* mutation status in TCGA non-small cell lung cancer database.

Supplementary Figure 6 Mutational signatures extracted from the aggregated NSCLC dataset. **(A)** The mutational activities of corresponding extracted mutational signatures (Signature 2, 4, 6, 7, 16, 26, and unmatched, named as COSMIC signature). **(B)** The mutational activities of corresponding mutational signatures showed in pie chart. **(C)** The mutational signature 2 and 4 in NSCLC cohort was associated with higher tumor mutation burden.

Supplementary Figure 7 Mutational patterns of recurrently mutated lung cancer genes, Genomic Instability associated genes and LRPs gene family in relation to *LRP1B* mutation in the pooled NSCLC patients. **(A)**. Mutation rates per megabase stratified by synonymous and non-synonymous mutations. **(B)**. The left panel is mutation frequency and the middle panel depicts genes mutation patterns across each cases with different mutation types color coded differently. Clinical features of immune response status, age, gender and stage displays in bottom. Immune response related genes were highlighted in bold.

Supplementary Figure 8 GSEA was performed on RNA-seq from pretreatment tumors in the melanoma cohorts by using the hallmark gene sets. Enrichment plots show increased enrichment of the immune-related gene set and decreased expression of Complement Cascade gene set in the *LRP1B* mutant samples.

Supplementary Figure 9 Kaplan-Meier survival analysis stratified by LRP1B mutation status in melanoma and NSCLC cohort from TCGA database.

Supplementary Table 1 Detailed sequencing information of each cohort.

Supplementary Table 2 Clinical and genome characteristics of each cancer patient.

Supplementary Table 3 Mutational signatures and activities in melanoma patients.

Supplementary Table 4 Mutational signatures and activities in NSCLC patients.

## Abbreviations

LRP1B: Low-density lipoprotein receptor-related protein 1B
ICB: immune check-point blockade
CTLA-4: Cytotoxic T-lymphocyte protein 4
PD-1: Programmed cell death protein 1
NSCLC: non-small cell lung cancer
TMB: tumor mutation burden
NB: neoantigene burden
HR: hazards ratio
OR: odds ratio
TCGA: The Cancer Genome Atlas
COSMIC: Catalog of Somatic Mutations in Cancer
GSEA: gene set enrichment analysis
LRPs: Low-density lipoprotein receptor-related protein families
SKCM: cutaneous melanoma
ESCC: esophagus squamous-cell carcinoma.
