# Corrigendum: Association of *LRP1B* Mutation With Tumor Mutation Burden and Outcomes in Melanoma and Non-small Cell Lung Cancer Patients Treated With Immune Check-Point Blockades

**DOI:** 10.3389/fimmu.2019.01523

**Published:** 2019-07-02

**Authors:** Hao Chen, Wei Chong, Qian Wu, Yueliang Yao, Min Mao, Xin Wang

**Affiliations:** ^1^Clinical Epidemiology Unit, Qilu Hospital of Shandong University, Jinan, China; ^2^Key Laboratory of Cancer Prevention and Therapy of Tianjin, Department of Epidemiology and Biostatistics, National Clinical Research Center for Cancer, Tianjin Medical University Cancer Institute and Hospital, Tianjin, China; ^3^Key Laboratory of Cancer Prevention and Therapy, Department of Breast Cancer Pathology and Research Laboratory, National Clinical Research Center for Cancer, Tianjin Medical University Cancer Institute and Hospital, Tianjin, China; ^4^Department of Respiratory Medicine, Central Hospital of Zibo, Zibo, China; ^5^Institute of Pathology and Southwest Cancer Center, Southwest Hospital, Army Medical University (Third Military Medical University), Chongqing, China; ^6^Key Laboratory of Tumor Immunopathology, Ministry of Education of China, Institute of Pathology and Southwest Cancer Center, Southwest Hospital, Third Military Medical University (Army Medical University), Chongqing, China; ^7^Department of Epidemiology and Biostatistics, First Affiliated Hospital, Army Medical University, Chongqing, China

**Keywords:** *LRP1B*, melanoma, NSCLC, tumor mutation burden, immunotherapy, mutation signatures

In the published article, there was an error in affiliation “1.” Instead of “Shandong Academy of Clinical Medicine, Shandong Provincial Hospital Affiliated to Shandong First Medical University, Jinan, China,” it should be “Clinical Epidemiology Unit, Qilu Hospital of Shandong University, Jinan, China.”

The authors apologize for this error and state that this does not change the scientific conclusions of the article in any way. The original article has been updated.

