# Supplementary figures and images for: Association of LRP1B Mutation With Tumor Mutation Burden and Outcomes in Melanoma and Non-small Cell Lung Cancer Patients Treated With Immune Check-Point Blockades
Source: Front Immunol. 2019 May 21;10:1113. doi: 10.3389/fimmu.2019.01113 (PMC6536574; doi:10.3389/fimmu.2019.01113)

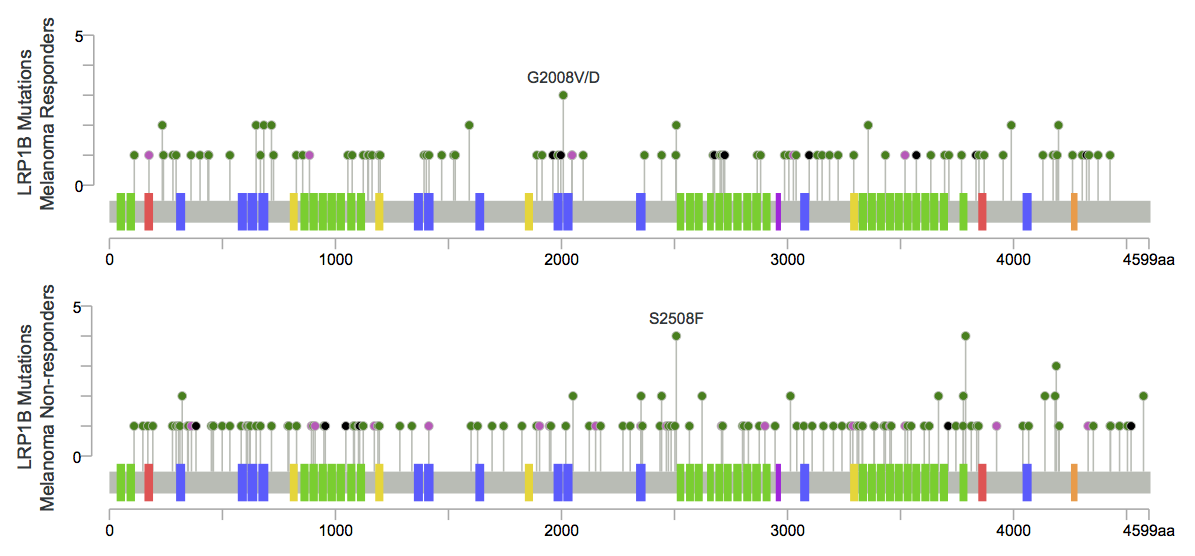

Supplement: Supplementary Figure 1 — Lollipop plot shows the distribution of LRP1B mutations in melanoma responder cohort and non-responder cohort. [file Image_1.TIFF]

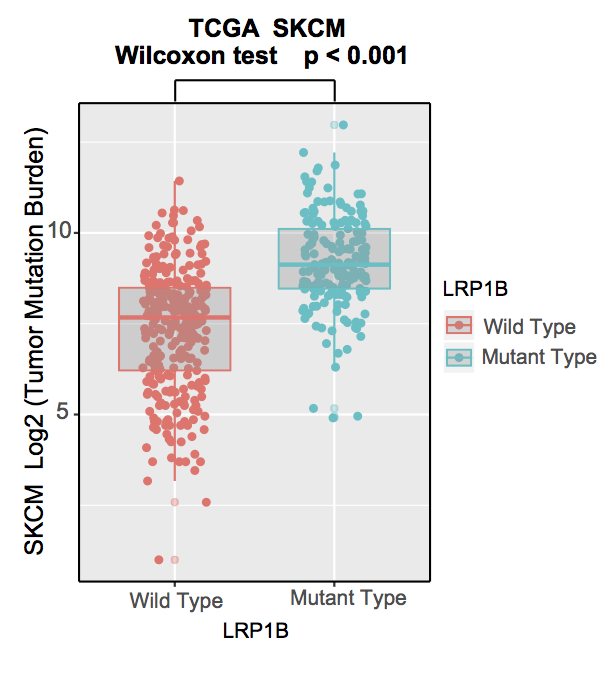

Supplement: Supplementary Figure 2 — Tumor mutation burden(TMB) associated with LRP1B mutation status in TCGA skin melanoma database. [file Image_2.TIFF]

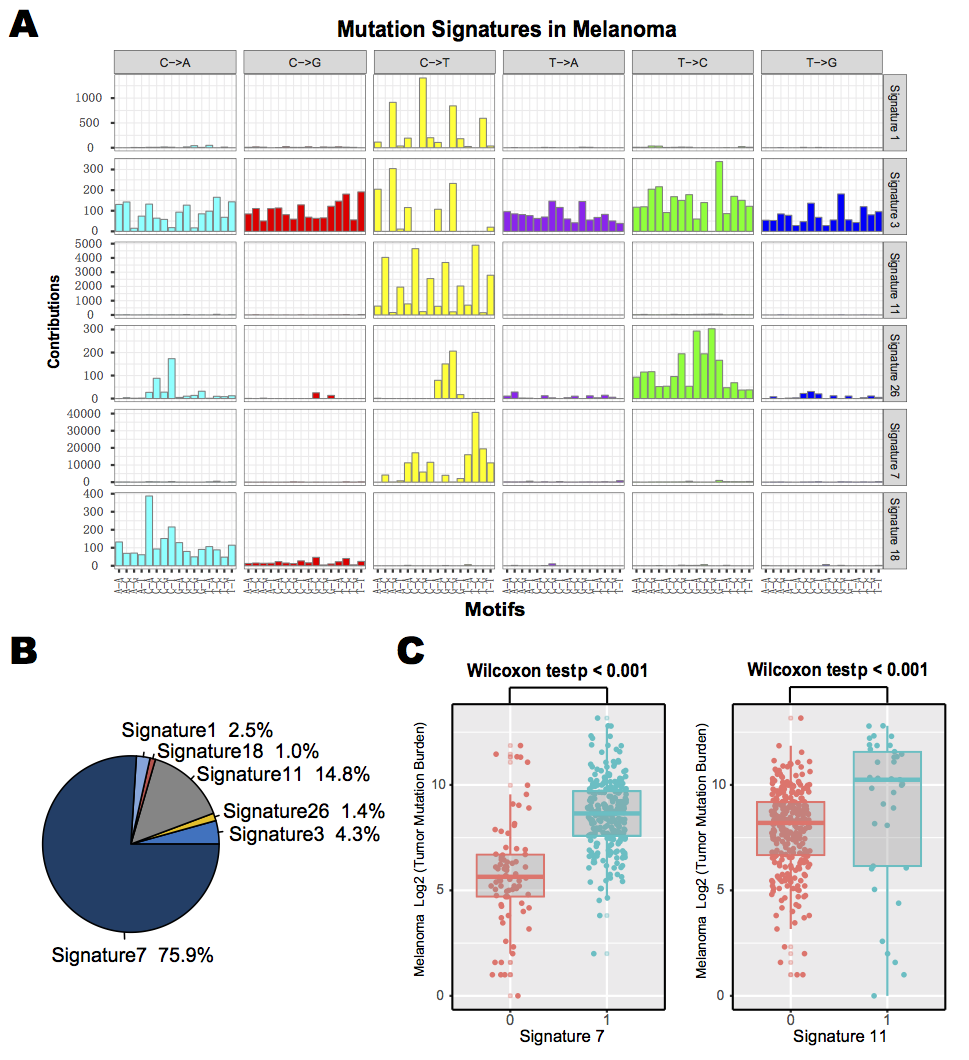

Supplement: Supplementary Figure 3 — Mutational signatures extracted from the aggregated melanoma dataset. (A) The mutational activities of corresponding extracted mutational signatures (Signature 1, 3, 7, 11, 18, 26, named as COSMIC signature). (B) The mutational activities of corresponding mutational signatures showed in pie chart. (C) The mutational signature 7 and 11 in melanoma cohort was associated with higher tumor mutation burden. [file Image_3.TIFF]

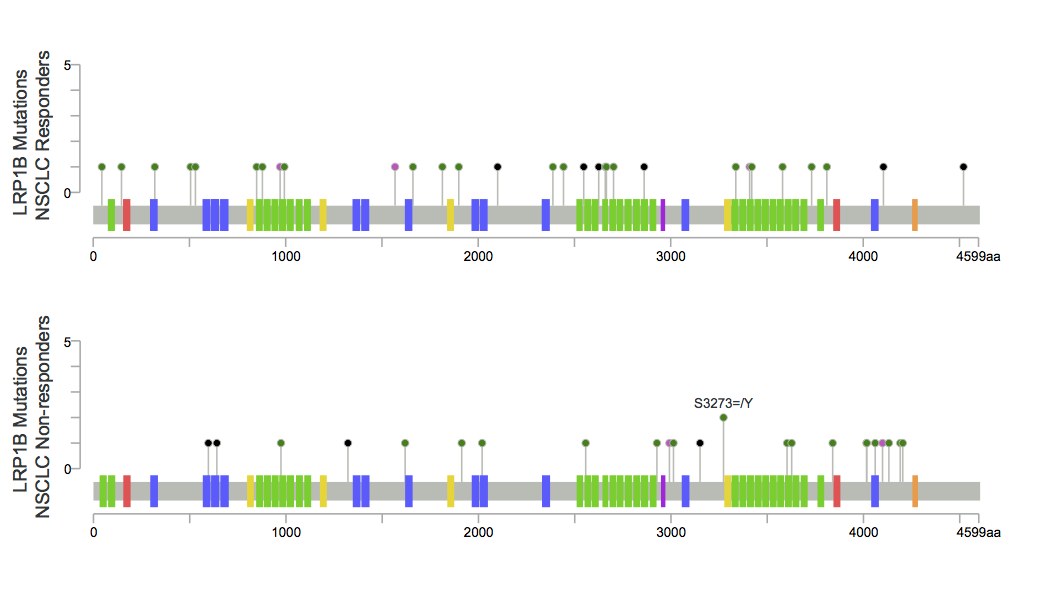

Supplement: Supplementary Figure 4 — Lollipop plot shows the distribution of LRP1B mutations in non-small cell lung cancer responder and non-responder cohort. [file Image_4.TIFF]

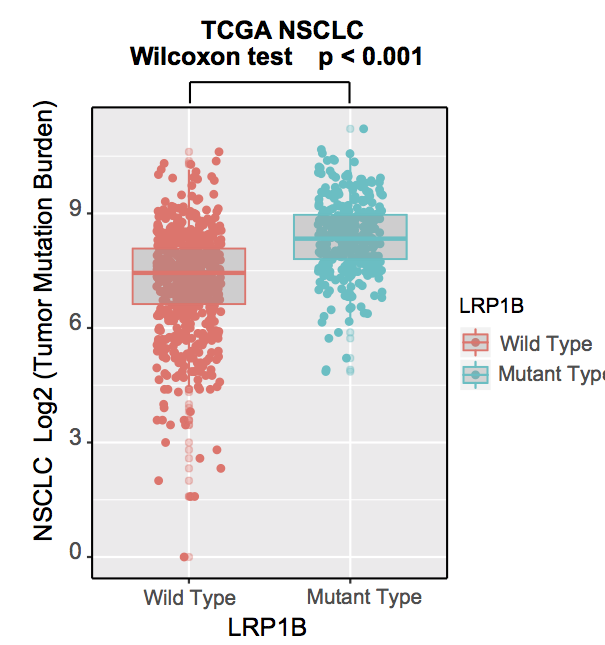

Supplement: Supplementary Figure 5 — Tumor mutation burden(TMB) associated with LRP1B mutation status in TCGA non-small cell lung cancer database. [file Image_5.TIFF]

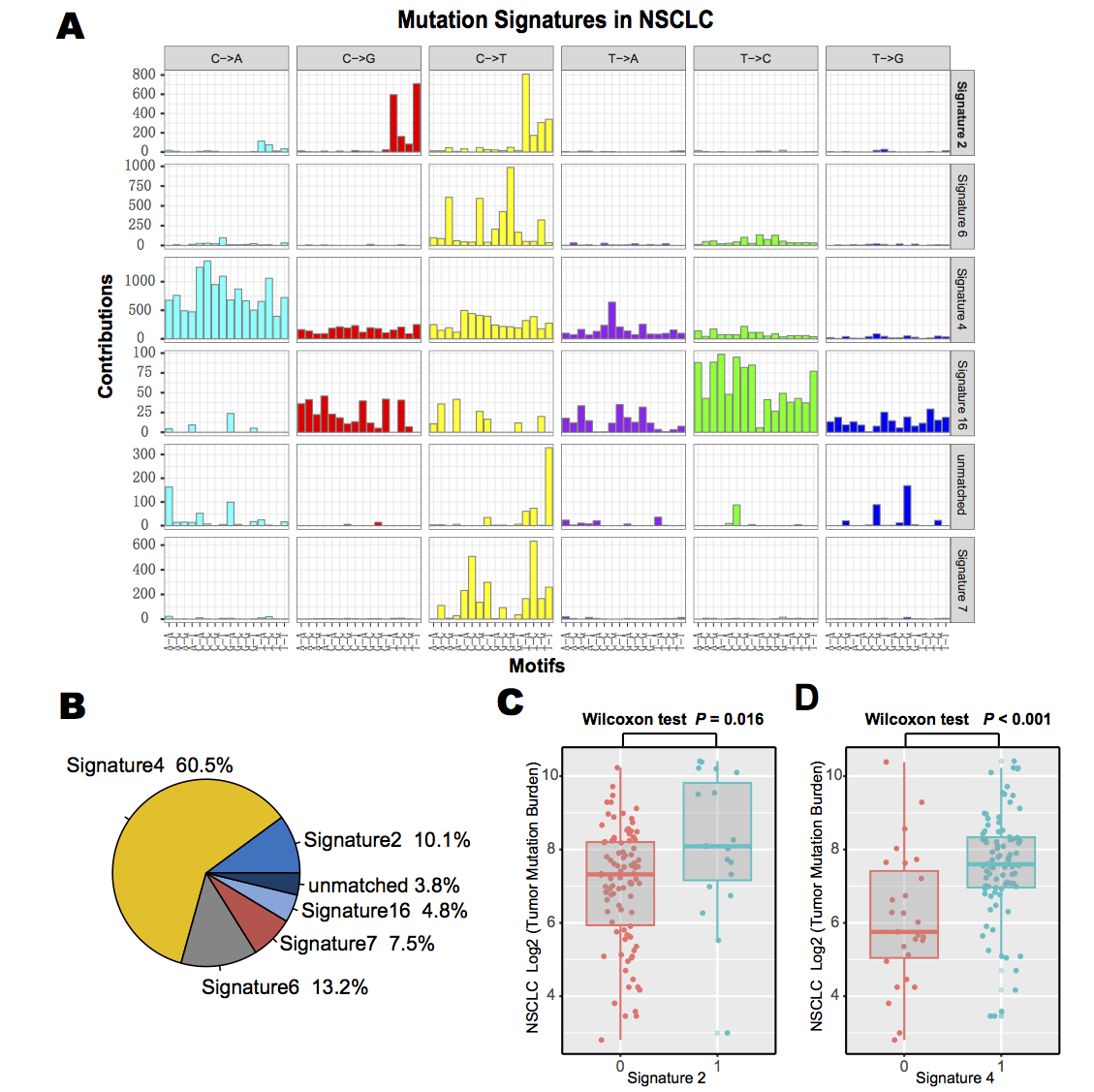

Supplement: Supplementary Figure 6 — Mutational signatures extracted from the aggregated NSCLC dataset. (A) The mutational activities of corresponding extracted mutational signatures (Signature 2, 4, 6, 7, 16, 26, and unmatched, named as COSMIC signature). (B) The mutational activities of corresponding mutational signatures showed in pie chart. (C) The mutational signature 2 and 4 in NSCLC cohort was associated with higher tumor mutation burden. [file Image_6.TIFF]

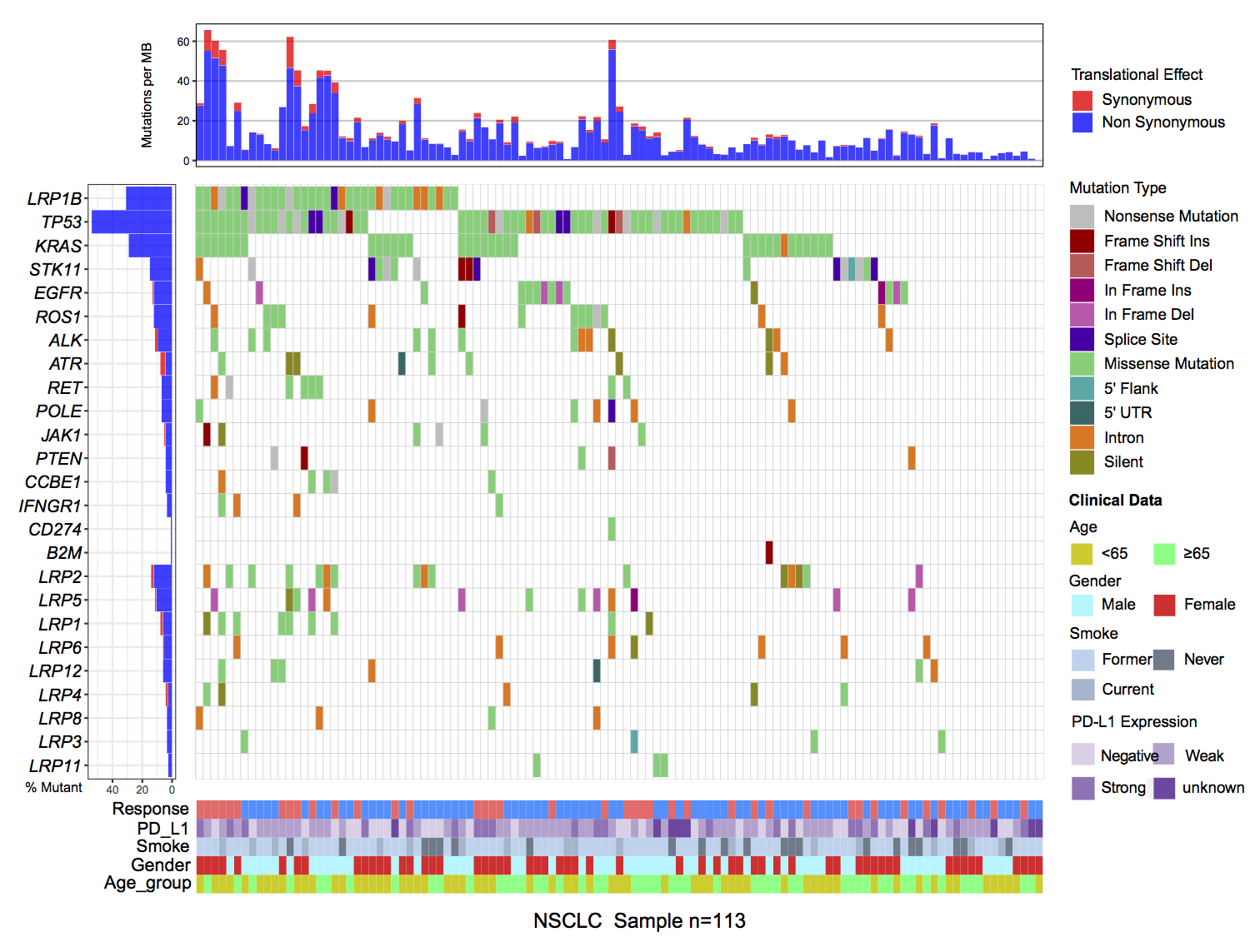

Supplement: Supplementary Figure 7 — Mutational patterns of recurrently mutated lung cancer genes, Genomic Instability associated genes and LRPs gene family in relation to LRP1B mutation in the pooled NSCLC patients. (A). Mutation rates per megabase stratified by synonymous and non-synonymous mutations. (B). The left panel is mutation frequency and the middle panel depicts genes mutation patterns across each cases with different mutation types color coded differently. Clinical features of immune response status, age, gender and stage displays in bottom. Immune response related genes were highlighted in bold. [file Image_7.TIFF]

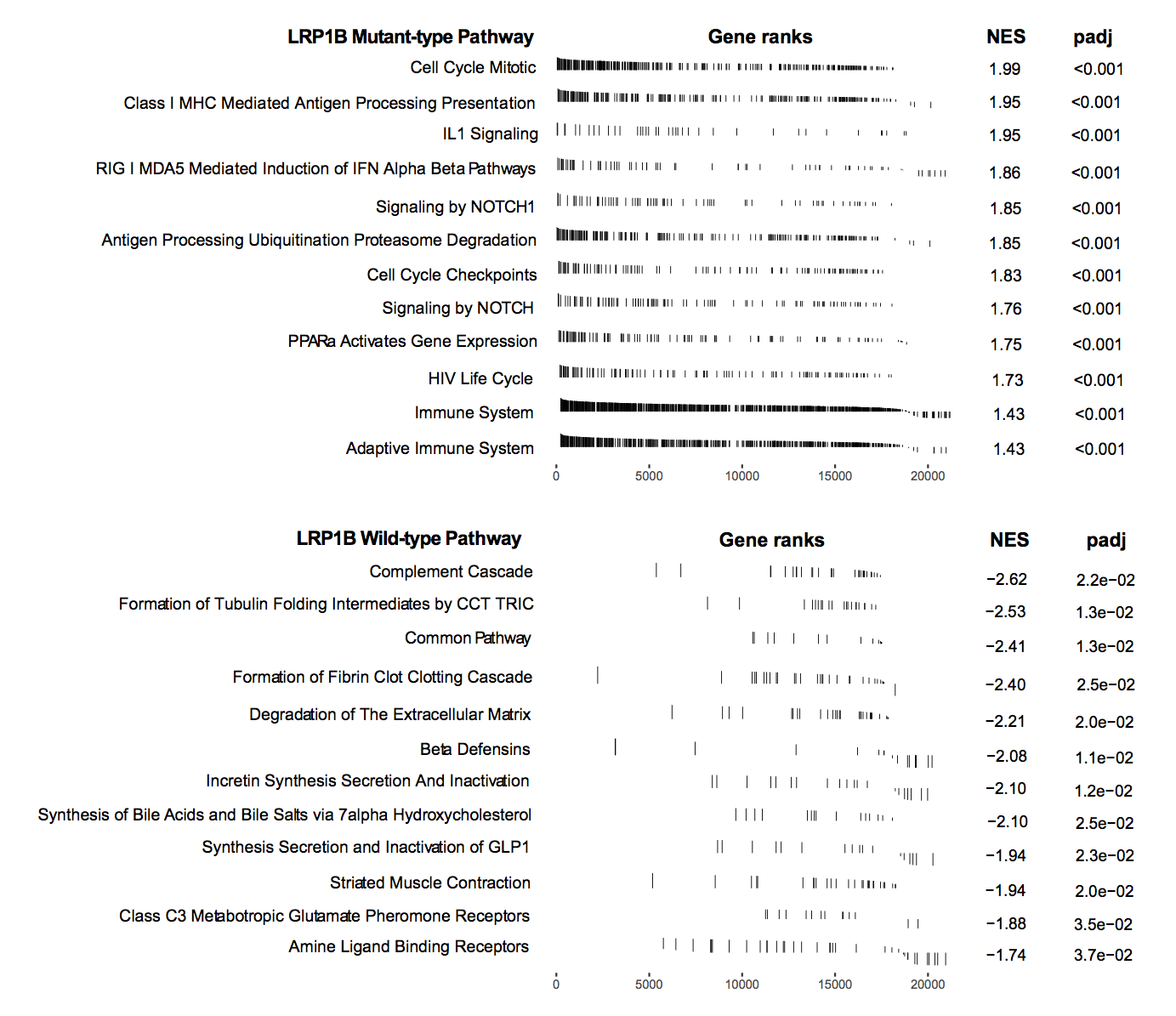

Supplement: Supplementary Figure 8 — GSEA was performed on RNA-seq from pretreatment tumors in the melanoma cohorts by using the hallmark gene sets. Enrichment plots show increased enrichment of the immune-related gene set and decreased expression of Complement Cascade gene set in the LRP1B mutant samples. [file Image_8.TIFF]

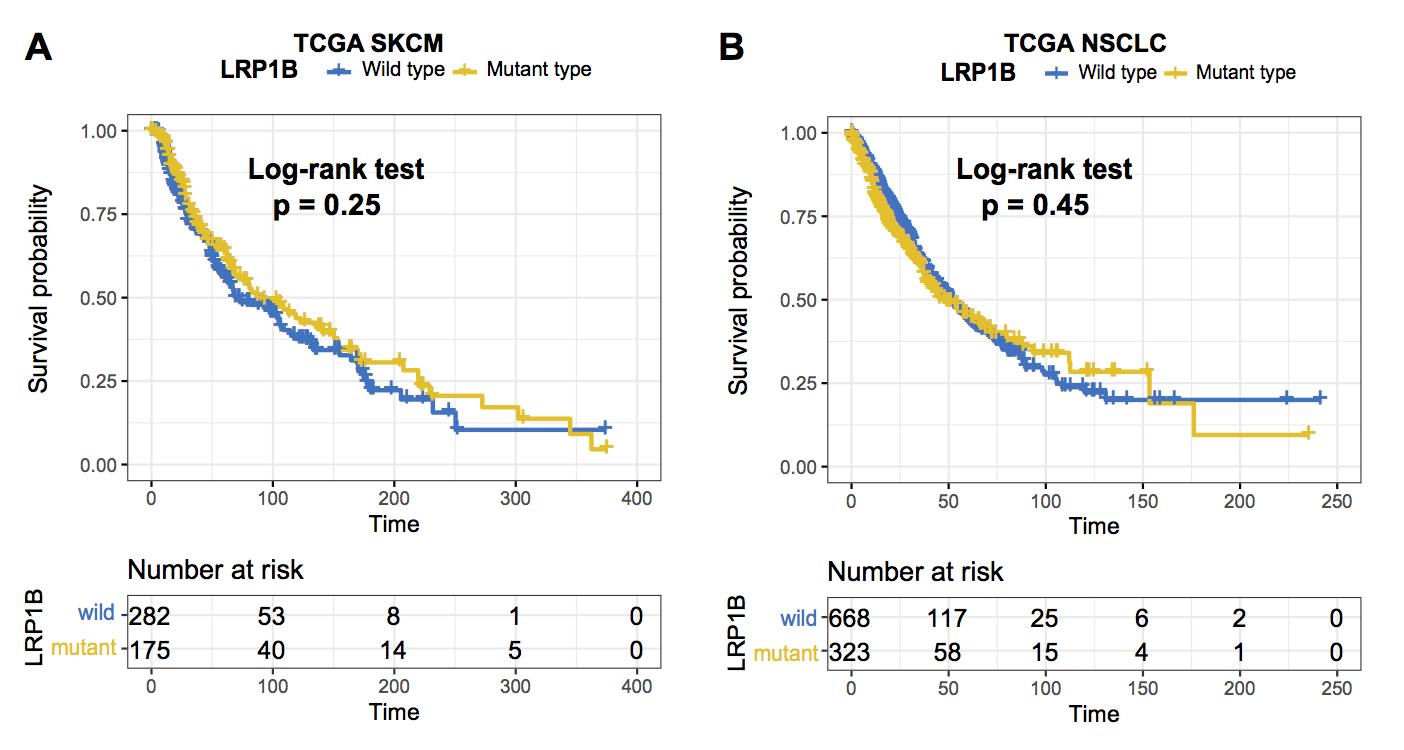

Supplement: Supplementary Figure 9 — Kaplan-Meier survival analysis stratified by LRP1B mutation status in melanoma and NSCLC cohort from TCGA database. [file Image_9.TIFF]
